# Working beyond SPA and the trajectories of cognitive and mental health of UK pensioners: Do gender, choice, and occupational status matter?

**DOI:** 10.1007/s10433-021-00644-4

**Published:** 2021-07-29

**Authors:** Baowen Xue, Manacy Pai, Minhao Luo

**Affiliations:** 1 Department of Epidemiology and Public Health, University College London, London, UK; 2 Department of Sociology, Kent State University, Kent, OH USA; 3 Health Insurance Division, China Pacific Life Insurance Company, Shanghai, China

**Keywords:** Cognition, Depression, ELSA, Involuntary retirement, Voluntary retirement

## Abstract

We assessed the association between work status beyond state pension age (SPA) and the long-term trajectories of cognitive and mental health for men and women separately, and the extent to which this relationship is conditioned by their occupational status and whether the choice to retire or continue working is voluntary or involuntary. Data are pensioners (aged between SPA and SPA + 9) from the English Longitudinal Study of Ageing waves 4 (2008/09) through 9 (2018/19). The analytic sample includes 959 men and 1217 women when considering cognitive outcomes and 1131 men and 1434 women when evaluating depression. Findings based on growth curve models reveal that, compared to women who retired at SPA and without any particular reason, their peers who retired due to frailing health reported a more precipitous decline in memory over time (coefficient = −0.10). However, analysis stratified by occupation shows that this association between ill-health retirement and long-term memory decline was concentrated among older women of the highest occupational status. We also found that men who retired or worked past SPA voluntarily reported a better baseline verbal fluency and were less likely to report depression over time (coefficient for work = 0.80; coefficient for retired = 0.87). Women who worked past SPA voluntarily were less likely to report depression at baseline (OR = 0.53). Policies that extend work life should offer older people more personal control over decision surrounding retirement.

**Supplementary Information:**

The online version contains supplementary material available at 10.1007/s10433-021-00644-4.

## Introduction

Given the shrinking size of working age compared to post-working age persons, most governments across Europe have implemented policies to disincentivize early exits from the labour force and instead, raised the state pension age (SPA) for retirement (Komp 2018). Through prolonged employment, such reforms are expected to mend the flailing pension systems. However, if older adults are unable to work until the raised SPA or if prolonged work impairs health, the fiscal burden might simply shift from post-retirement pensions to other parts of social insurance, including health care. Understanding the association between working past SPA and cognitive and mental health, as such, remains critical to policies on work and retirement.

Drawing on the English Longitudinal Study of Ageing (ELSA), a nationally representative sample of people aged 50 years and over in England, we aim to contribute to this end in four ways: First, while most extant research is limited to understanding either the overall effect of retirement on health (i.e. health status change pre-and post-retirement) or the health repercussions of early retirement (e.g. Atalay and Barrett 2014; Nishimura et al. 2018), we assess the association between working beyond SPA and cognitive and mental health. Second, we explore the extent to which the health consequences of working past SPA are conditioned by whether the decision to do so is voluntary or involuntary. Third, given existing gender disparities in experiences related to work, health, and life in general (Calasanti and Slevin 2001), we assess the above associations for men and women separately. Lastly, given that the health effects of both, employment and early retirement are linked to work type and conditions (Calvo et al. 2013), we examine the extent to which the associations between working past SPA and cognitive and mental health are moderated by occupational status.

### Paid work and cognitive and mental health

Through employment, most people acquire new skills, adapt to changing workplace demands, meet new people and engage in social interactions, all requiring the use of several high-ordered cognitive processes that help maintain cognitive reserve (Stern 2012) and protect against cognitive decline (Bjelajac et al. 2019). Retirement may reduce everyday opportunities to engage in cognitively complex activities increasing the risk of cognitive decline (Bianchini and Borella 2016; Bonsang et al. 2012). Retirement, also, may result in mental distress given that, according to role theory, paid work offers social and psychological resources both of which protect against stressful circumstances, alleviate distress, and improve mental health (Thoits 2011; Wang et al. 2011). Unless prepared for alternate social roles and activities in post-retirement years, those who exit the workforce may lack the structure, social interactions, and predictability that accompany most paid work. Alternatively, according to the psychosocial-environmental hypothesis, retirement could support both, cognitive and mental health to the degree that it removes work stress and work-family conflicts, (Andel et al. 2016; Axelrad et al. 2017) and frees up time to focus on health (van der Heide et al. 2013) and other mentally revitalizing activities, such as volunteering, which may facilitate a more stable transition to retirement (Henning et al. 2016).

### Retirement timing and health

The association between retirement and health may be conditioned by whether retirement is deemed early, on time, or delayed based on one’s age at the time of this transition (Börsch-Supan and Jürges 2009). Chronological age, which is used to allocate social ranking, establishes expectations, which in turn prescribe a “social timetable” for all major life course transitions, including retirement (Neugarten et al. 1965). Based on the *cultural-institutional* hypothesis (Dannefer et al. 2011), transitions that occur “on time” match the existing cultural scripts and as such, yield better health outcomes compared to ones that transpire “off-time”. When a transition is “off” time or defies the so-called social clock, individuals are likely deprived of the otherwise expected or “anticipatory socialization” related to that transition. For instance, while retiring early liberates an individual from work-related tedium and working past retirement age ensures sustained financial benefits, both, the early and delayed retirement may deprive older adults of the “shared” experiences related to this transition—unless their peers also happen to retire simultaneously. While empirical findings on how timing conditions the health impact of retirement remain conflicted (Calvo et al. 2013), some studies have found more positive mental and physical health outcomes for those whose retirement matches the culturally expected timing associated with this transition (van Solinge and Henkens 2007).

### Gender and occupational class

Given their varying employment histories, opportunities, and experiences, health ramifications of working past SPA may vary by gender. Women and men have different workforce attachments. Most men consider work to have a central role in their lives whereas women are equally invested in non-paid work roles (Quick and Moen 1998; McMunn 2015). Prolonged paid work, especially working past SPA, may more negatively affect older women if it is combined with other non-paid work roles (such as caregiving); retirement, as such, may provide greater protection against cognitive decline and improve mental health for women. Alternatively, the gendered division of labour affects not just employment experiences but also retirement (Calasanti and Slevin 2001). That is, while men often reap full-time leisure as a prize for a lifespan of employment (Barnes and Parry 2004), women often end up doing the same or added amount of housework after retirement (Quick and Moen 1998). Continued employment, consequently, could provide older women with sustained social-psychological resources, which could positively affect both, their cognitive and mental health (Easterlin 2003).

Working past SPA may also render differential cognitive and mental health effects for older workers based on their occupational status. In particular, blue-collar jobs that are characterized by high job strain, constant supervision, and lack of creativity and autonomy render workers more vulnerable to mental and cognitive distress (Karasek 1979; Ravesteijn et al. 2018). Individuals retiring early from physically strenuous jobs report better health and cognition whereas, for their peers in less physically demanding jobs, early retirement translates into reduced health (Mazzonna and Peracchi 2017). Prolonged work also may result in differential health outcomes for workers in higher versus lower occupational statuses because while the former may revel in their work, the latter often are forced to extend working for financial reasons. Additionally, jobs that require creativity and complexity—typically, ones in higher occupational bracket—may help older adults retain function due to greater “cognitive reserves” (Stern 2012). Jobs that lack complex and creative tasks, unfortunately, preclude the opportunity to invest in and build the human capital necessary to protect against cognitive decline typically associated with ageing.

### Choice in decision to work or retire

Regardless of the type and status of employment, personal choice in whether to retire or continue working reflects personal control over one’s immediate environment. Control theories (Zarit et al. 2003) suggest that important life transitions, such as retirement, over which there is no control (e.g. involuntary retirement or forced prolonged work) may compromise health (Szinovacz and Davey 2005). Expectedly then, previous studies do find individuals who retire involuntarily report more adverse mental health effects relative to their counterparts who retire voluntarily (Gallo et al. 2006; van Solinge and Henkens 2007). Most extant studies, however, are limited to research on either the overall effect of retirement on health or the health repercussions of early retirement.

## Data

Data are from the English Longitudinal Study of Ageing (ELSA). ELSA is a cohort study on the health, economics, and welfare of the ageing population in England, which aims to represent people aged 50 and over living in private households in England. The initial samples were drawn in 2002. A follow-up survey, which was conducted every two years to form a wave, has been repeated 9 times thus far. Ethical approval for all the ELSA waves was secured from the National Research and Ethics Committee.

### Sample

We utilized ELSA waves 4 (2008/09) through 9 (2018/19) for this study. Wave 4 is the first wave including questions about the reasons why participants still work beyond SPA and wave 9 is the most recent one available (Barnes et al. 2020). Because we aim to assess the health impact of work status of pensioners, participants who did not reach SPA at wave 4 were excluded. The SPA was 65 for male and 60 for female participants at wave 4 (2008/09). In other words, males younger than 65 and females younger than 60 at wave 4 were not included in our study (Bozio et al. 2010). The upper age limit of the participants was also restricted, 74 years for men and 69 years for women; this decision reflected the negligible proportion of individuals remaining in the workforce beyond these ages (Di Gessa et al. 2018). People who ‘never worked’ or did not report the reason to retire/work and respondents without any valid response on outcome variables between waves 4 and 9 were excluded. Also excluded were participants with missing data on the conceptually relevant covariates. Finally, persons who reported having dementia at baseline were excluded when assessing cognitive function as the outcome. The final analytic sample includes 959 men and 1217 women when considering cognitive outcomes and 1131 men and 1434 women when evaluating depression. Figure S1 in online Supplementary Information displays the process of sample selection.

### Measures

#### Outcome variables

The outcome variables are cognitive function and psychological distress over 10 years of follow-up in ELSA. Cognitive function is assessed using verbal episodic memory and verbal fluency. To assess verbal episodic memory, participants listened to a list of 10 common words and were asked to recall as many as possible, both immediately and after a short delay. The score scale of memory is from 0 to 20 which combines the score of immediate and delayed recall with a higher score indicating better memory (Murre et al. 2013). To test verbal fluency, which also is reflective of executive functioning, participants were asked to name as many animals as possible within a minute, with a score range of 0–55 with higher scores indicative of better performance (Shao et al. 2014). Depressive symptoms, used to assess psychological distress, were measured by the abbreviated 8-item version of the Centre for Epidemiological Studies Depression Scale (CES-D; Radloff 1977). Participants with a score greater than 4 are considered as having high depressive symptoms (Ní Mhaoláin et al. 2012). Memory and depression were repeatedly measured at each wave between waves 4 through 9, and verbal fluency was repeatedly measured at waves 4, 5, 7, 8, and 9.

#### Independent variable

The main independent variable was work status combined with the motivation driving the decision to either work or retire. It was measured at wave 4. While participants could offer multiple motivations, they were also asked the main motivation for work/retirement, which we used in this study. The distribution of specific reasons that motivated the decision to either continue working or retire is available in online Supplementary Information Table S1. We grouped these reasons into four types: reached retirement age, own ill health, voluntary, and involuntary reasons. Then, we grouped participants into 6 categories by combing motivation and work status: in work after SPA and voluntary reason for work (labelled as ‘work and voluntary’); in work after SPA and involuntary reason for work (labelled as ‘work and involuntary’); retired and voluntary reason for retiring (labelled as ‘retired and voluntary’); retired and involuntary reason for retiring (labelled as ‘retired and involuntary’); retired and own ill-health reason (labelled as ‘retired and ill health’); retired and the reason is reached retirement age (labelled as ‘retired and SPA’) (Di Gessa et al. 2018).

#### Covariates

We included whether work status remains the same in the follow-up (no; yes) and marital status (‘married/cohabit’, ‘single’, ‘divorced/separated’ and ‘widowed’) as time-varying variables. Other covariates were measured at baseline (wave 4). Age was centred by SPA for men and women, separately. Ethnicity included white or non-white. Childhood social class was measured by father’s occupation at age 14, including ‘Manager/Professional’, ‘Non-manual’ ‘Manual’ and ‘Other’. The highest educational qualification was categorized as degree (International Standard Classification of Education-ISCED level 6), higher education below degree (ISCED level 4 and 5), A level (ISCED level 3), O level (ISCED level 2), lower than O level/foreign/other, and no qualification. Occupational class (before retirement) was measured by the National Statistics Socio-economic Classification three-class version (managerial/professional, intermediate, and routine/manual). Household wealth (quintiles) and number of children were also included. Health covariates comprised the presence of limiting long-standing illness (no; yes, not limiting; yes, limiting), and any limitations with the activities of daily living (no; yes) measured by ADL and IADL. Baseline depression and objectively measured grip strength were adjusted when assessing cognitive health. These covariates were chosen due to their well-documented relationship with work status and cognition and depression in the literature (e.g. Rice et al. 2011; Sternäng et al. 2016; Jorm 2000; Xue et al. 2018).

To minimize the practice effects of the cognitive tests, the ELSA questionnaire used four different and validated 10-word lists to access delayed recall in each wave. Additionally, we included the square root of the number of previous visits (e.g. 0, 1, 1.4, 1.7…) in the regression models to account for re-test effects (Vivot et al. 2016; Romero Starke et al. 2019).

#### Statistical method

We employed growth curve models (also known as multilevel models). The growth curve model included respondents if they have at least one wave of response on the health outcome between wave 4 and 9. Linear growth curve models were applied for continuous outcomes (memory and fluency), and logistic growth curve models were used for the binary depression variable. People who retired and the reason was “reached retirement age” (‘retired and SPA’) were used as the reference group.

A ‘time’ variable was generated in the study to represent the follow-up time. This time variable ranges from 0 (wave 4) to 5 (wave 9), and every unit increase in this time variable indicates a 2-year increase in the follow-up time. The coefficient of this time variable shows the slope of individual trajectories of cognition or depression over time. A quadratic term of time was included in the model to represent the nonlinear trajectories of outcomes. Interaction between the independent variable and time was included in the model to assess the long-term impact of work status/motivation. Analyses were conducted for episodic memory, verbal fluency, and depression, respectively. An interaction between baseline age and time was also included for cognition to reflect the complex relationship between age and cognition (no significant interaction for depression, and thus was not included).

Considering the close linkage between occupational class and work status/motivation, we also assessed whether the occupational class is an effect modifier by including an interaction between work status/motivation and occupational class in the models.

Sensitivity analysis by excluding early retirement before SPA was conducted.

## Results

Table 1 shows the descriptive characteristics of older male and female participants in our study. In our sample, women were on average 5.4 years younger than men. Women were more likely to work after SPA than men, either involuntarily (12.4% vs. 5.2%) or voluntarily (23.4% vs. 14.6%). For both men and women, ‘retired and voluntary’ was the most common reason for retirement, followed by ‘retired and ill health’. Compared to men, women were 9% less likely to live with a partner, have a degree qualification (16% vs. 20%), be in a management and professional occupational class, but women were more likely to come from a managerial/professional and non-manual childhood social class (40% vs. 33%). Women had fewer children than men. The distribution of race was comparable between men and women, with 98% being white. Women, on average, had 1.7 higher score of memory and 1 higher score of verbal fluency than men; women also, however, were more likely to report depression and had lower grip strength.

**Table 1:** Characteristics of men and women in this study^a^

	Women (*n* = 1217) %	Men (*n* = 959)%
*Work status and motivation*		
Retired and SPA	13.06	18.35
Retired and ill health	17.09	20.44
Retired and involuntary	12.08	15.75
Retired and voluntary	21.94	25.65
Work and involuntary	12.41	5.21
Work and voluntary	23.42	14.60
*Ethnicity*		
White	98.11	97.60
Non-white	1.89	2.40
*Marital status*		
Single	4.27	5.32
Married/cohabit	67.13	76.54
Divorced/separated	14.79	8.45
Widowed	13.80	9.70
*Education*		
Degree	16.19	20.23
Higher eeducation below degree	13.48	17.41
A Level	7.81	6.47
O Level	22.43	17.31
Lower than O Level/foreign/other	12.33	11.68
No qualification	27.77	26.90
*Occupational class*		
Managerial/professional	28.51	37.43
Intermediate	28.92	22.42
Routine/manual/other	42.56	40.15
*Father’s occupation*		
Manager/Professional	20.13	15.33
Non-manual	19.88	17.41
Manual	37.39	40.77
Other	22.60	26.49
*Household income*		
Lowest quintile	13.80	15.33
2	18.32	15.75
3	20.79	20.33
4	21.04	22.73
Highest quintile	26.05	25.86
*Number of children*		
0	12.08	11.26
1	14.13	11.57
2	36.48	38.58
3	22.43	22.21
4 or more	14.87	16.37
*Limitations*		
Yes	22.35	25.55
No	77.65	74.45
*Depression*		
No	83.98	91.03
Yes	16.02	8.97
*Long-standing illness*		
None	46.43	41.71
Yes and limiting	30.40	32.85
Yes and not limiting	23.17	25.44
Mean age, yr (SD)	64.11 (2.97)	69.49 (2.86)
Mean grip strength, kg (SD)	23.14 (6.15)	36.36 (8.66)
Mean memory (SD)	11.31 (3.27)	9.66 (3.13)
Mean verb fluency (SD)	21.55 (6.60)	20.60 (6.63)

^a^n is based on the sample used for memory analysis

**Fig. 1:**
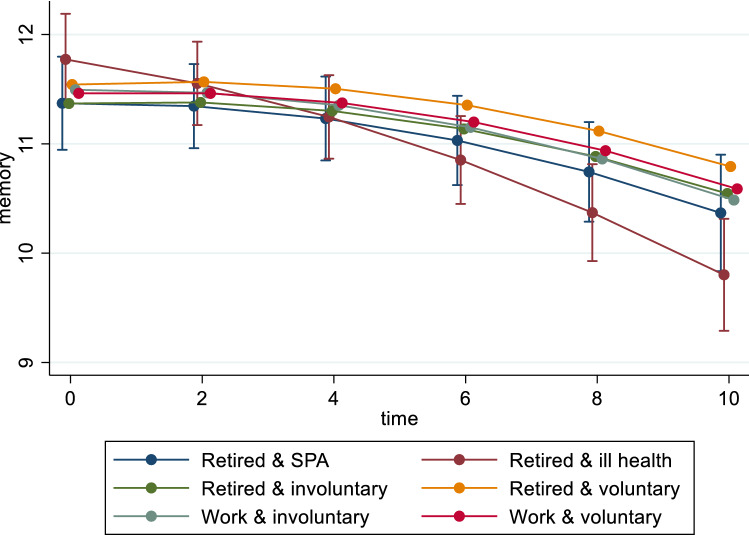
Work status beyond SPA and the trajectory of memory for women

Table 2 shows the associations between work status after SPA and memory for men and women, separately. Women in different groups of work status, either in work or retired, for voluntary reasons or not, all had similar memory at baseline (i.e. similar intercepts). However, during the 10-year follow-up between waves 4 and 9, women who retired for own ill health showed a faster rate (slope) of memory decline over time than women in the ‘retired and SPA’ group (trajectories are shown in Fig. 1). The coefficient of the interaction between ‘retired and ill health’ and time variable was −0.10 (95%CI: −0.17, −0.02), suggesting that, every year, for women who retired for own ill health their memory scores declined by 0.10 more than for their peers in the ‘retired and SPA’ group. While statistically significant, this effect might be marginal considering that the average memory score of women is 11. Women in other work status groups show similar rates of memory decline in the follow-up as women in the ‘retired and SPA’ group (i.e. no interaction between independent variable and time). Men in different work statuses after SPA did not show differences in their memory either in the baseline or in the follow-up (trajectories are shown in online Supplementary Information Figure S2).

**Table 2:** Association between work status beyond SPA and the trajectory of memory by gender

	Women (*n* = 1217)		Men (*n* = 959)	
	Coef	95% CI	Coef	95% CI
*Work status and motivation*				
Retired and SPA	Ref.		Ref.	
Retired and ill health	0.40	−0.18, 0.98	−0.08	−0.63, 0.46
Retired and involuntary	−0.002	−0.60, 0.59	−0.50	−1.06, 0.05
Retired and voluntary	0.17	−0.35, 0.69	0.29	−2.05, 0.79
Work and involuntary	0.12	−0.48, 0.73	0.54	−0.25, 1.34
Work and voluntary	0.09	−0.45, 0.63	0.16	−0.42, 0.74
*Work status and motivation* × *Time*				
Retired and SPA	Ref.		Ref.	
Retired and ill health	−0.10**	−0.17, −0.02	−0.06	−0.14, 0.03
Retired and involuntary	0.02	−0.06, 0.10	0.03	−0.05, 0.12
Retired and voluntary	0.03	−0.04, 0.09	0.01	−0.06, 0.08
Work and involuntary	0.03	−0.08, 0.08	0.03	−0.09, 0.15
Work and voluntary	0.01	−0.06, 0.09	0.03	−0.06, 0.12
*Baseline age*	−0.13***	−0.18, −0.07	−0.07*	−0.13, −0.01
*Marital status*				
Single	Ref.		Ref.	
Married/cohabit	−0.31	−1.03, 0.41	1.12**	0.34, 1.90
Divorced/separated	−0.30	−1.05, 0.44	1.16**	0.31, 2.01
Widowed	−0.23	−0.97, 0.50	1.27**	0.43, 2.10
*Father’s occupation*				
Manager/professional	Ref.		Ref.	
Non-manual	0.25	−0.18, 0.68	−0.36	−0.88, 0.16
Manual	−0.15	−0.54, 0.25	−0.15	−0.61, 0.32
Other	−0.15	−0.58, 0.28	0.11	−0.40, 0.62
*Education*				
Degree or higher	Ref.		Ref.	
Higher education below degree	−0.79***	−1.29, −0.29	−1.10***	−1.59, −0.60
A Level	−0.32	−0.93, 0.28	−1.55***	−2.22, −0.87
O Level	−0.46 ^ŧ^	−0.95, 0.03	−1.01***	−1.53, −0.50
Lower than O/foreign/other	−1.02***	−1.58, −0.47	−1.31***	−1.92, −0.71
No qualification	−1.81***	−2.31, −1.30	−2.05***	−2.58, −1.52
*Occupational class*				
Managerial/profession	Ref.		Ref.	
Intermediate	−0.08	−0.46, 0.31	−0.23	−0.65, 0.19
Routine/manual/other	−0.41*	−0.80, −0.02	−0.68**	−1.08, −0.28
*Ethnicity*				
White	Ref.		Ref.	
Non-white	−0.92^ŧ^	−1.93, 0.09	−1.71^t^	−2.67, −0.75
*Illness*				
None	Ref.		Ref.	
Yes and limiting	−0.13	−0.51, 0.25	−0.26	−0.64, 0.13
Yes and not limiting	−0.07	−0.42, 0.27	0.04	−0.33, 0.40
*Baseline depression*				
Without depression	Ref.		Ref.	
With depression	−0.44*	−0.83, −0.05	−0.50^ŧ^	−1.04, 0.04
*Change work status*				
No	Ref.		Ref.	
Yes	−0.05	−0.33, 0.23	−0.43*	−0.82, −0.04
*Household income*				
Lowest quintile	Ref.		Ref.	
2	0.36	−0.13, 0.85	−0.11	−0.64, 0.42
3	0.51*	0.01, 1.01	−0.02	−0.55, 0.50
4	0.90**	0.39, 1.41	−0.05	−0.58, 0.49
Highest quintile	1.00***	0.48, 1.52	0.14	−0.43, 0.70
*Number of children*				
0	Ref.		Ref.	
1	0.21	−0.36, 0.77	−0.56	−1.25, 0.13
2	0.25	−0.25, 0.74	−0.25	−0.85, 0.36
3	0.12	−0.41, 0.64	−0.25	−0.88, 0.38
4 or more	0.05	−0.52, 0.62	−0.22	−0.89, 0.45
*Limitations*				
Yes	Ref.		Ref.	
No	0.43*	0.05, 0.81	0.23	−0.15, 0.62
Grip strength	0.05***	0.03, 0.07	0.03**	0.01,0.05
Practice effect	1.17***	0.79, 1.54	0.95**	0.58, 1.31
Time	0.05	−0.03, 0.14	−0.05	−0.15, 0.06
Time × Time	−0.01***	−0.02, −0.01	−0.01**	−0.02, −0.003
Baseline age × Time	−0.01**	−0.02, −0.003	−0.01	−0.02, 0.002
*Random effect*				
Variance of time	0.02	0.02, 0.03	0.03	0.02, 0.05
Variance of constant	4.09	3.67, 4.57	3.44	3.00, 3.93

^ŧ^*p* < 0.1 *p < 0.05 **p < 0.01 ***p < 0.001

In term of effect modifications, there was an interaction between work status and occupational class for women’s memory trajectories (*p* < 0.05). Analysis stratified by occupation shows that the association between ill-health retirement and long-term memory decline was concentrated among older women of the managerial/professional (highest) occupational status (Table 3). Coefficient of the interaction between ‘retired & ill health’ and time is −0.18 (95%CI:−0.33, −0.03).

**Table 3:** Association between work status beyond SPA and the trajectory of memory among women in the managerial/professional (highest) occupational class (n = 347)

	Coef	95% CI
*Work status and motivation*		
Retired and SPA	Ref.	
Retired and ill health	−0.10	−1.27, 1.07
*Work status and motivation* × *Time*		
Retired and SPA	Ref.	
Retired and ill health	−0.18*	−0.33, −0.03

*p < 0.05

While work status after SPA was not associated with long-term trajectory of verbal fluency either for men or women, men who retired or continued working for voluntary reasons reported a better baseline verbal fluency. Results and predicted trajectories of verbal fluency with men and women’s work status are shown in Table S2 and Figure S3-S4 in online Supplementary Information. No effect modifier role of occupational class was found for vernal fluency (results are not shown in tables).

Table 4 shows the association between work status beyond SPA and the long-term trajectory of depression by gender. Women who continued to work voluntarily were 43% (OR: 0.53; 95%CI: 0.29, 0.97) less likely to report depression than their peers in the ‘retired and SPA’ group at baseline, and this difference endured over time (trajectories are shown in online Supplementary Information Figure S5). However, women who retired for voluntary reasons were more likely to report depression in the follow-up, although this effect was only marginally significant (0.05 < *p* < 0.1).

**Table 4:** Association between work status beyond SPA and the trajectory of depression by gender

	Women (*n* = 1434)		Men (*n* = 1131)	
	OR	95% CI	OR	95% CI
*Work status and motivation*				
Retired and SPA	Ref.		Ref.	
Retired and ill health	1.49	0.88, 2.55	2.05^ŧ^	0.93, 4.52
Retired and involuntary	0.88	0.47, 1.62	2.12^ŧ^	0.91, 4.94
Retired and voluntary	0.62	0.35, 1.12	1.06	0.43, 2.59
Work and involuntary	1.04	0.56, 1.94	0.85	0.23, 3.17
Work and voluntary	0.53*	0.29, 0.97	1.25	0.42, 3.70
*Work status and motivation* × *Time*				
Retired and SPA	Ref.		Ref.	
Retired and ill health	1.10^ŧ^	0.99, 1.22	0.90	0.78, 1.03
Retired and involuntary	1.09	0.97, 1.23	0.93	0.81, 1.08
Retired and voluntary	1.11^ŧ^	0.99, 1.24	0.87^ŧ^	0.75, 1.01
Work and involuntary	1.01	0.89, 1.15	1.16	0.93, 1.43
Work and voluntary	1.02	0.90, 1.15	0.80*	0.65, 0.99
*Baseline age*	0.96	0.92, 1.01	0.98	0.91, 1.05
*Marital status*				
Single	Ref.		Ref.	
Married/cohabit	0.80	0.39, 1.63	−0.36*	0.14, 0.92
Divorced/separated	1.09	0.52, 2.26	0.70	0.25, 1.93
Widowed	1.50	0.72, 3.12	1.43	0.54, 3.79
*Father’s occupation*				
Manager/professional	Ref.		Ref.	
Non-manual	0.94	0.61, 1.46	1.10	0.54, 2.26
Manual	1.10	0.74, 1.63	0.81	0.42, 1.57
Other	0.89	0.58, 1.36	0.91	0.45, 1.83
*Education*				
Degree or higher	Ref.		Ref.	
Higher education below degree	0.84^t^	0.50, 1.42	1.13	0.54, 2.36
A Level	0.59	0.31, 1.10	2.14^ŧ^	0.89, 5.19
O Level	1.01	0.62, 1.64	1.43	0.68, 2.98
Lower than O Level/foreign/other	0.77	0.44, 1.34	0.98	0.42, 2.28
No qualification	0.91	0.55, 1.50	1.03	0.49, 2.16
*Occupational class*				
Managerial/profession	Ref.		Ref.	
Intermediate	1.52*	1.03, 2.27	1.21	0.69, 2.15
Routine/manual/other	1.55*	1.05, 2.28	1.14	0.67, 1.95
*Ethnicity*				
White	Ref		Ref	
Non-white	1.10^ŧ^	0.99, 1.22	3.65**	1.41, 9.47
*Illness*				
None	Ref		Ref.	
Yes and limiting	2.32***	1.64, 3.28	2.49**	1.48, 4.20
Yes and not limiting	1.14	0.79, 1.65	1.27	0.73, 2.22
*Baseline depression*				
Without depression	Ref		Ref	
With depression	−0.50	−1.04, 0.04	−0.44*	−0.83, −0.05
*Change work status*				
No	Ref		Ref	
Yes	1.13	0.70, 1.82	0.80	0.32, 1.95
*Household income*				
Lowest quintile	Ref		Ref	
2	0.86	0.57, 1.30	0.60	0.32, 1.12
3	0.76	0.49, 1.17	0.60	0.32, 1.12
4	0.52**	0.33, 0.83	0.53^ŧ^	0.27, 1.03
Highest quintile	0.50	0.31, 0.82	0.39*	0.19, 0.81
*Number of children*				
0	Ref		Ref	
1	0.71	0.42, 1.23	1.13	0.47, 2.73
2	0.80	0.50, 1.29	0.80	0.36, 1.76
3	0.94	0.57, 1.56	0.86	0.37, 2.00
4 or more	0.91	0.54, 1.56	0.85	0.35, 2.04
*Limitations*				
Yes	Ref		Ref	
No	0.45***	0.33, 0.62	0.33***	0.21, 0.53
*Time*	0.73	0.65, 0.84***	0.98	0.83, 1.17
*Time* × *Time*	1.02***	1.01, 1.03	1.01	1.00, 1.02
Random effect				
*Variance of time*	0.05	0.03, 0.08	0.04	0.01, 0.10
*Variance of constant*	1.32	0.85, 2.05	2.49	1.54, 4.03

^ŧ^*p* < 0.1, *p < 0.05, **p < 0.01 ***p < 0.001

**Fig. 2:**
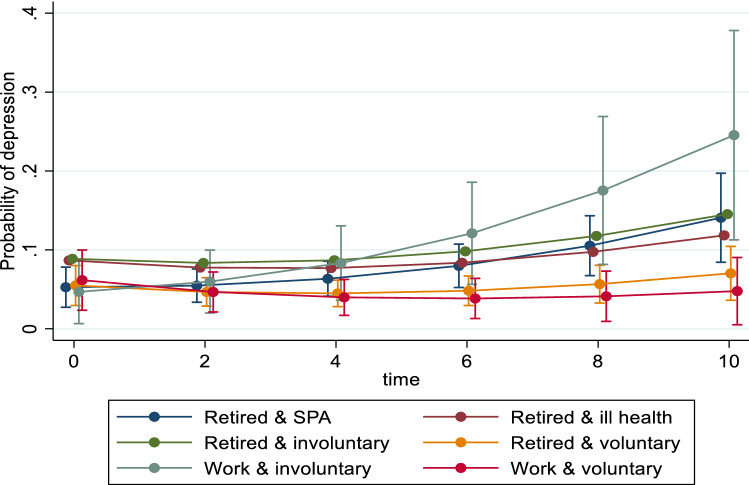
Work status beyond SPA and the trajectory of depression for men

Compared to men in the group of ‘retired and SPA’, men who retired for involuntary reasons or own ill health were about 2 times more likely to report depression at baseline (borderline significance), and this difference endured over time. Men who work for voluntary reasons showed a lower rate (slope) of developing depression than their peers in the ‘retired and SPA’ group in the follow-up (coefficient for work and voluntary × time = 0.80, 95%CI: 0.65, 0.99). Trajectories are shown in Fig. 2. This suggests a beneficial association between working voluntarily past SPA and mental health in the long-term for men. Similar long-term pattern was observed for men who retired voluntarily, although the association appears to be weaker (coefficient = 0.87) and only marginally significant (0.05 < *p* < 0.1).

Results from the sensitivity analysis (Table S3–S5 in online Supplementary Information) are consistent with our main results, and additionally, the sensitivity analysis shows that women who retired due to ill health reported a more precipitous decline in verbal fluency over time.

## Discussion

We examined the association between work status beyond SPA and the long-term trajectories of cognitive and mental health for men and women separately, and the extent to which this relationship is conditioned by occupational status and whether the choice to retire or continue working is voluntary or involuntary. We found that women who retired due to ill health reported a more precipitous decline in memory over time, however, this association concentrated among older women of the highest occupational status. Our study also revealed that compared to men who retired at SPA, those who retired or worked past SPA voluntarily reported a better baseline verbal fluency and were less likely to report depression over time. Women who worked beyond SPA voluntarily were less likely to report depression at baseline.

### Work, retirement, and cognitive health

Compared to women who retired at SPA and without any particular reason, their peers who retired due to frailing health reported a more precipitous decline in memory over time. Over time, exiting the workforce may reduce structured opportunities to engage in cognitively complex activities increasing the risk of cognitive decline (Bianchini and Borella 2016; Bonsang et al. 2012; Xue et al. 2018). Ill health also may limit mobility, prevent leisure pursuits and social activities, and instead force consolidating of daily activities around health problems (Charmaz 1991). Put simply, ill health that propelled retirement may compromise social and health mechanisms needed to ensure cognitive performance in later life.

However, the memory decline associated with ill-health retirement is concentrated among those in the highest occupational status. On one hand, our finding is consistent with a recent study by Xue and colleagues (2018) who find that higher occupational status is protective against cognitive decline while individuals continue to work, but the “protective effect” ceases upon retirement. Relative to lower-status jobs, those of higher occupational status, which involve more intellectually complex, challenging, and creative tasks protect individuals against cognitive decline (Schooler et al. 1999). As such, according to the “use it or lose it” hypothesis, exiting from a higher-status job may mean the loss of cognitive resources necessary for healthy cognition. Moreover, given that professionals are more likely to reap intrinsic benefits from their work (Sass 2016), ill-health retirement also may represent a loss of psychological resources (e.g. sense of worth), negatively influencing memory over time.

On the other hand, based on the cognitive reserves hypothesis (Stern 2012), persons in highly complex and creative jobs are expected to enjoy a “protracted” protective effect of having worked in jobs that require higher-ordered cognitive processes (e.g. problem-solving; strategic thinking). Consequently, those in the highest occupational grade are expected to avail established (i.e. pre-retirement) cognitive mechanisms or/and acquire new ones to manage memory fluctuations (Steffener and Stern 2012). We encourage future scholarship to assess exact factors (e.g. perceptions of occupational prestige and stigma associated with retiring from a higher-status job) that amplify cognitive decline in this particular group of women.

Women of all other work statuses—be it still working or retired either voluntarily or involuntarily—reported comparable memory function both, at baseline and over time relative to their counterparts who retired at SPA without citing any particular reason. The lack of statistically meaningful differences between women of the other diverse work groups is consistent with conclusions from a recent review on the long-term repercussions of retirement on cognition. Based on 29 longitudinal studies, Alvarez-Bueno and colleagues (2020) concluded that retirement does not negatively impact overall cognition among older adults and only slightly adversely affects their memory functioning. In our study, the lack of statistically significant differences between women of diverse work statuses may reflect the fact that the women in our sample are much older while most existing studies include relatively younger (i.e. young-old) group of women as they are focused on assessing the cognitive health impact of early retirement as opposed to testing the cognitive health effects of working past SPA. For similar reasons, we speculate, our study also fails to find statistically meaningful differences in the memory function of older men of diverse work statuses, either at baseline or over time. We found that men who retired or worked past SPA voluntarily reported a better baseline verbal fluency, and this difference endured over time. Verbal fluency indicates crystallized cognitive abilities, and the baseline differences may reflect a selection into self-controlled work transitions rather than an effect of retiring or working past SPA.

### Work, retirement, and psychological health

Relative to their peers who retired at SPA, older men who continued working past SPA voluntarily were less likely to report depression over time, and this effect holds regardless of the occupational status. Employment is a source of not just economic resources, like income and health insurance, but social resources (e.g. social support) and psychological assets, namely sense of mastery and self-esteem (e.g. Wickrama et al. 1997). Such economic, social, and psychological resources, in turn, protect against stress and maintains mental health (Thoits 2011). Research shows that older adults who remain active and engaged in personally and socially fulfilling activities, such as paid work, report less distress and better mental health (Hao 2008; Wethington et al. 2000). Our finding that working past SPA is positively consequential for mental health of older men is of significance for both, individuals and rapidly ageing societies. The implication is for us to create and maintain work environments that are conducive for the mental health of older workers who wish to continue working past SPA. Interestingly, a similar beneficial mental health association was observed for men who retired for voluntary reasons, although the association appears to be weaker and only marginally significant.

Women who work past SPA voluntarily are also less likely to report depression at baseline, and this difference endured over time. Important to note, however, is that women who retired voluntarily report an increased (albeit, marginally significant) risk of developing depression over time, a finding which contradicts what we find for men in our sample. One possible explanation is that the advantages attached to retirement may cancel out by the disadvantages typically associated with this transition. For instance, while retirement liberates women from work-family conflict, it also triggers negative attitudes about retirement given that the current cohort of older women still have the same (or in fact, added) household obligations after retirement as they did before retiring (Calasanti 1996; Quick and Moen 1998).

The overall findings here are indicative of three general inferences. First, health in later life hinges less on whether a person is retired on time or working past SPA and more on the choice surrounding the decision to retire or continue working. This is particularly the case for older men, and this could reflect gendered socialization and gender variations in meanings attached to formal social roles. Second, the complexity surrounding retirement demands that we continue to assess the impact of this transition on health within the context of individual characteristics, gender being one of them. And, finally, the health effect of retirement or extended work life is far from static; in fact, our findings suggest that it is more likely to shift over time. It is the unfolding of cognitive and mental functioning over time that is likely to portray a picture that is closer to the realities surrounding work, retirement, and health.

## Limitations and future directions

Our findings, we caution, need to be inferred within the context of important limitations. First, the ambiguous association between retirement and health, at least, partially can be attributed to the insufficient account of health selection bias, that is, that poor health may be a cause, not a consequence, of workforce exit (Bound et al. 1999). As such, when assessing cognitive decline, we excluded those respondents who reported having dementia at baseline and treated as a separate category those individuals who reported retiring due to ill health. However, reverse causality still is possible and discussed in a number of studies (e.g. Behncke 2012; Bonsang et al. 2012; Coe and Lindeboom 2008) that employ advanced research designs to tackle the endogeneity problem related to the link between health and retirement. Nonetheless, most extant work including ours lacks any perfect solution to this issue and partly because most observed effects related to work, health, and retirement are tied both to established social-structural contexts or/and variations within specific sub-groups of workers/retirees. Moreover, the association between retirement and health remains susceptible to a multitude of unobservable factors, such as personality and genetic pre-dispositions or/and family-level processes (Calvo et al. 2013).

Second, although we have adjusted for occupational class and tested interactions between work and occupational statuses, specific work conditions before retirement, namely job strain, opportunities for meaningful social interaction, and creativity, were not adjusted in the models, as these variables were only measured among those who were currently employed at the time of the interview. Future scholarship should consider the questions of what changes emerge in occupations over time? Which occupations translate into phased retirement or bridge jobs? How may the job traits within different occupations affect work behaviour and expectations, retirement timing in the future cohorts of older workers, and ultimately their cognitive and mental health over time. Identifying characteristics of occupations associated with pre-SPA could point out specific areas in need of policy reform.

Additionally valuable would be to discern how couples and families, in addition to individuals, respond to pension reforms. Retirement as a transition has increasingly become a couples’ transition as opposed to being limited to an individual decision (Hospido 2015). This may be particularly true for women. Women, on an average, have fewer financial resources and women of older cohorts, especially, are relatively less attached to labour force than men; consequently, their decision to retire may be even more influenced by their partners’ decisions surrounding work and retirement (van der Horst et al. 2017). Interestingly, the latest research (Bertogg et al. 2021) based on data from the European Union Statistics reveals that women’s likelihood of retiring is increased even if they are the main earner in the household, which suggests that women are more likely to compensate for their non-traditional income by retiring earlier. Given these findings and the continued increase in women’s labour force participation (Gehringer and Klasen 2017), retirement likely will remain a matter of joint determination and as such, pension policies related to work and retirement are most likely to succeed if we can discern not just how individuals, but couples and families respond to them.

Third, growth curve models can be estimated in the presence of partially missing data (including individuals with data from only one measurement occasion) if the missing data mechanism can be assumed to be missing completely at random or missing at random (Raudenbush and Bryk 2002). We think the missing at random assumption is reasonable in our case, as the observed data captured key confounding influence, e.g. long-standing illness and other socio-economic factors which related to both attrition and the outcome of interest. That said, nonignorable missingness still is possible, and given that some respondents dropped out of the sample due to death or poor health, the generalizability of the findings remains limited.

Fourth, we grouped several reasons of work/retirement into four broad categories (reached retirement age, own ill health, voluntary, and involuntary reasons). Future research should investigate the heterogeneity within groups and assess which reason and circumstance related to retirement is particularly consequential for older adults’ health. This strand of future work may also consider, in addition to work status, the health repercussions of work histories. And, lastly, given that the welfare reform policies vary across countries, future studies should explore the relationships we are assessing in our study within a cross-cultural context to evaluate whether the associations between later life work transitions and health extend across nations and as such, across varying socio-institutional contexts.

## Conclusion

Continuing to assess the cognitive and mental health impact of working beyond SPA is important given that policy reform to extend working lives, to a large extent, is predicated on the assumption that today’s older adults, unlike their predecessors, are in much better health. If working beyond the current SPA improves cognitive and mental health, this finding would bolster policy efforts to further prolong work lives and perhaps, stimulate concrete ways to engage and facilitate older workers into more productive careers. Conversely, if working after SPA is reflective of a decline in cognitive and mental well-being, delaying retirement would be problematic both, for the individual and overall economy given the rising health care costs accrued from declining health. Moreover, understanding the relevance of personal choice and motivation in conditioning the health consequences of retirement or prolonged employment can inform family members, practitioners, and policymakers as they pinpoint opportunities for improving retirement-related decisions and guide the choices of a future generation of older workers and retirees.

## Supplementary Information

Below is the link to the electronic supplementary material.

Supplementary file1 (DOCX 123 kb)
