# Supplementary material for: Working beyond SPA and the trajectories of cognitive and mental health of UK pensioners: Do gender, choice, and occupational status matter?
Source: Eur J Ageing. 2021 Jul 29;19(3):423–36. doi: 10.1007/s10433-021-00644-4 (PMC9424372; doi:10.1007/s10433-021-00644-4)
Supplement: Supplementary file 1 — Supplementary file1 (DOCX 123 kb) [file 10433_2021_644_MOESM1_ESM.docx]

n=11050 at wave 4

Restricted age:

Female 60-69 Male 65-74

(excluded n=7667)

n=3383

(F:1945, M:1438)

Excluded 20 never work

n=3363

(F:1930, M:1433)

Excluded 591 without giving work/ retired reasons

n=2772

(F:1530, M:1242)

Excluded participants without any valid response between wave 4 and 9

(excluded n=56 for cognition, n=60 for depression)

After excluding 536 missing data on covariates

n(F)=1217; n(M)=959

Cognition

n(F)=1509

n(M)=1203

Excluded 4 diagnosed dementia at baseline

After excluding 147 missing data on covariates

n(F)=1434; n(M)=1131

Depression

n(F)=1510

n(M)=1202

**Figure S1** Flow chart of sample selection

**Table S1 Distribution of reasons for retiring/working above SPA by subsamples of each work status.**

| **Work status & motivation** | **% of total (n=2176)** | **% of subsamples** |
| --- | --- | --- |
| Retired & SPA | 15.4 |  |
| Retired & ill health | 18.57 |  |
| Retired & involuntary | 13.69 | % within ‘Retired & involuntary’ |
| *Ill health of a relative/friend* |  | *32.89* |
| *Made redundant/dismissed/had no choice* |  | *65.77* |
| *Could not find another job* |  | *1.34* |
| Retired & voluntary | 23.58 | % within ‘Retired & voluntary’ |
| *To spend more time with partner/ family* |  | *20.27* |
| *To enjoy life while still young and fit* |  | *25.34* |
| *Fed up with job and wanted a change* |  | *14.23* |
| *To retire at the same time as husband/w* |  | *7.02* |
| *To retire at a different time to husband/w* |  | *0.19* |
| *To give the young generation a chance* |  | *0.97* |
| *Life event (re)married/moved house/spou* |  | *0.39* |
| *Could afford to* |  | *0.19* |
| *Offered reasonable financial terms* |  | *31.38* |
| Work & involuntary | 9.24 | % within ‘Work & involuntary’ |
| *Could not afford to retire earlier* |  | *55.72* |
| *Improve pension/financial position* |  | *44.28* |
| Work & voluntary | 19.53 | % within ‘Work & voluntary’ |
| *Did not know what to do after stopping w* |  | *4.24* |
| *Enjoyed job/working* |  | *68* |
| *To keep fit and active* |  | *19.53* |
| *To retire at the same time as husband/w* |  | *3.53* |
| *Persuaded by employer to stay on* |  | *4.71* |

**Table S2** Association between work status beyond SPA and the trajectory of verbal fluency by gender.

|  | **Women (n=1217)** | | **Men (n=959)** | |
| --- | --- | --- | --- | --- |
|  | **Coef.** | **95% CI** | **Coef.** | **95% CI** |
| *Work status & motivation* |  |  |  |  |
| Retired & SPA | Ref |  | Ref |  |
| Retired & ill health | 0.22 | -0.99, 1.43 | 0.08 | -1.11, 1.27 |
| Retired & involuntary | -1.02 | -2.26, 0.22 | 0.55 | -0.67, 1.78 |
| Retired & voluntary | 0.15 | -0.94, 1.24 | 1.24* | 0.14, 2.33 |
| Work & involuntary | 0.49 | -0.78, 1.75 | 1.18 | -0.57, 2.94 |
| Work & voluntary | 0.39 | -0.73, 1.51 | 1.86** | 0.58, 3.15 |
| *Work status & motivation × Time* |  |  |  |  |
| Retired & SPA | Ref |  | Ref |  |
| Retired & ill health | -0.10 | -0.24, 0.05 | -0.002 | -0.16, 0.16 |
| Retired & involuntary | 0.02 | -0.13, 0.18 | 0.02 | -0.15, 0.19 |
| Retired & voluntary | 0.04 | -0.09, 0.18 | 0.10 | -0.04, 0.25 |
| Work & involuntary | -0.03 | -0.20, 0.14 | 0.11 | -0.13, 0.35 |
| Work & voluntary | -0.04 | -0.19, 0.11 | -0.03 | -0.06, 0.12 |
| *Baseline age* | -0.14^ŧ^ | -0.26, -0.03 | -0.07* | -0.21, 0.15 |
| *Marital status* |  |  |  |  |
| Single | Ref |  | Ref |  |
| Married/cohabit | 0.30 | -1.22, 1.83 | -0.02 | -1.77, 1.73 |
| Divorced/seperated | 0.47 | -1.12, 2.07 | -0.20 | -2.10, 1.70 |
| Widowed | 0.71 | -0.86, 2.28 | 0.51 | -1.35, 2.38 |
| *Father’s occupation* |  |  |  |  |
| Manager/professional | Ref |  | Ref |  |
| Non-manual | -0.19 | -1.09, 0.71 | -0.86 | -2.03, 0.31 |
| Manual | -0.41 | -1.24, 0.42 | -0.53 | -1.58, 0.52 |
| Other | -0.98* | -1.89, -0.06 | -0.50 | -1.65, 0.65 |
| *Education* |  |  |  |  |
| Degree or higher | Ref |  | Ref |  |
| Higher edu below degree | -1.51** | -2.57, -0.46 | -0.77 | -1.88, 0.35 |
| A Level | -1.46* | -2.74, -0.18 | -0.95 | -2.46, 0.57 |
| O Level | -1.62** | -2.65, -0.60 | -0.82 | -1.98, 0.34 |
| Lower than O Level/foreign/other | -2.70** | -3.88, -1.53 | -1.47* | -2.82, -0.11 |
| No qualification | -4.30** | -5.37, -3.22 | -2.78** | -3.97, -1.58 |
| *Occupational class* |  |  |  |  |
| Managerial/profession | Ref |  | Ref |  |
| Intermediate | -0.53 | -1.34, 0.29 | -0.32 | 1.26, 0.63 |
| Routine/manual/other | -0.67 | -1.50, 0.15 | -0.48 | -1.38, 0.43 |
| *Ethnicity* |  |  |  |  |
| White | Ref |  | Ref |  |
| Nonwhite | -4.72** | -6.84, -2.60 | -5.79 | -7.96, -3.62 |
| *Illness* |  |  |  |  |
| None | Ref |  | Ref |  |
| Yes & limiting | -0.38 | -1.17, 0.41 | 0.03 | -0.84, 0.90 |
| Yes & not limiting | 0.17 | -0.55, 0.90 | 0.14 | -0.68, 0.96 |
| *Baseline depression* |  |  |  |  |
| Without depression | Ref |  | Ref |  |
| With depression | 0.32 | -0.39, 0.90 | -0.65 | -1.87, 0.56 |
| *Change work status* |  |  |  |  |
| No | Ref |  | Ref |  |
| Yes | -0.05 | -0.33, 0.23 | 0.038* | -0.85, 0.93 |
| *Household income* |  |  |  |  |
| Lowest quintile | Ref |  | Ref |  |
| 2 | 0.38 | -065, 1.42 | -0.33 | -1.52, 0.86 |
| 3 | 1.07* | 0.02, 2.12 | 0.78 | -0.39, 1.95 |
| 4 | 0.87 | -0.20, 1.94 | -0.23 | -1.45, 0.98 |
| Highest quintile | 1.31* | 0.21, 2.41 | 1.26 | -0.01, 2.53 |
| *Number of children* |  |  |  |  |
| 0 | Ref |  | Ref |  |
| 1 | -0.47 | -1.67, 0.73 | 0.36 | -1.19, 1.92 |
| 2 | 0.23 | -0.82, 1.28 | -0.02 | -1.38, 1.33 |
| 3 | 0.10 | -1.01, 1.21 | 0.08 | -1.34, 1.50 |
| 4 or more | 0.64 | -0.56, 1.83 | 0.74 | -0.77, 2.24 |
| *Limitations* |  |  |  |  |
| Yes | Ref |  | Ref |  |
| No | 0.82* | 0.01, 1.63 | -0.05 | -0.92, 0.82 |
| *Grip strength* | 0.12** | 0.07, 0.16 | 0.07** | 0.03, 0.12 |
| *Practice effect* | 2.23** | 1.38, 3.07 | 1.63** | 0.74, 2.52 |
| *Time × Time* | 0.01 | 0, 0.2 | 0.004 | -0.01, 0.02 |
| *Baseline age × Time* | -0.02* | -0.04, -0.01 | -0.01 | -0.03, 0.004 |
| ^ŧ^ p<0.1 *p<0.05 **p<0.01 |  |  |  |  |

**SENSITIVITY ANALYSIS**

**Table S3** Associate between work status beyond SPA and the trajectory of memory by excluding early retirement

|  | **Women (n=1072)** | | **Men (n=816)** | |
| --- | --- | --- | --- | --- |
|  | **Coef.** | **95% CI** | **Coef.** | **95% CI** |
| **Work status & motivation** |  |  |  |  |
| Retired & SPA | Ref |  | Ref |  |
| Retired & ill health | 0.63* | 0.01, 1.25 | -0.10 | -0.68, 0.48 |
| Retired & involuntary | 0.06 | -0.58, 0.70 | -0.28 | -0.88, 0.32 |
| Retired & voluntary | 0.23 | -0.31, 0.77 | 0.46 | -0.07, 0.98 |
| Work & involuntary | 0.16 | -0.44, 0.76 | 0.55 | -0.24, 1.34 |
| Work & voluntary | 0.13 | -0.40, 0.67 | 0.14 | -0.44, 0.73 |
| **Work status & motivation × Time** |  |  |  |  |
| Retired & SPA | Ref |  | Ref |  |
| Retired & ill health | -0.12** | -0.20, -0.04 | -0.07 | -0.16, 0.02 |
| Retired & involuntary | -0.01 | -0.09, 0.08 | 0.01 | -0.08, 0.10 |
| Retired & voluntary | 0.02 | -0.05, 0.09 | 0.01 | -0.07, 0.09 |
| Work & involuntary | 0.00 | -0.09, 0.08 | 0.04 | -0.08, 0.16 |
| Work & voluntary | 0.01 | -0.06, 0.08 | 0.04 | -0.06, 0.13 |

*p<0.05 **p<0.01

**Table S4** Associate between work status beyond SPA and the trajectory of verbal fluency by excluding early retirement

|  | **Women (n=1072)** | | **Men (n=816)** | |
| --- | --- | --- | --- | --- |
|  | **Coef.** | **95% CI** | **Coef.** | **95% CI** |
| **status & motivation** |  |  |  |  |
| Retired & SPA | Ref |  | Ref |  |
| Retired & ill health | 0.56 | -0.74, 1.87 | 0.08 | -1.22, 1.38 |
| Retired & involuntary | -0.94 | -2.29, 0.40 | 0.39 | -0.96, 1.73 |
| Retired & voluntary | 0.39 | -0.74, 1.52 | 1.23* | 0.06, 2.40 |
| Work & involuntary | 0.46 | -0.81, 1.73 | 1.17 | -0.61, 2.96 |
| Work & voluntary | 0.34 | -0.79, 1.47 | 1.74** | 0.43, 3.05 |
| **Work status & motivation × Time** |  |  |  |  |
| Retired & SPA | Ref |  | Ref |  |
| Retired & ill health | -0.16* | -0.32, -0.01 | -0.03 | -0.20, 0.14 |
| Retired & involuntary | 0.01 | -0.16, 0.18 | -0.02 | -0.20, 0.16 |
| Retired & voluntary | 0.04 | -0.10, 0.18 | 0.15 | -0.002, 0.30 |
| Work & involuntary | -0.02 | -0.19, 0.14 | 0.12 | -0.11, 0.36 |
| Work & voluntary | -0.04 | -0.18, 0.11 | -0.01 | -0.19, 0.16 |

*p<0.05 **p<0.01

**Table S5** Associate between work status beyond SPA and the trajectory of depression by excluding early retirement

|  | **Women (n=1262)** | | **Men (n=959)** | |
| --- | --- | --- | --- | --- |
|  | **OR** | **95% CI** | **OR** | **95% CI** |
| **Work status & motivation** |  |  |  |  |
| Retired & SPA | Ref |  | Ref |  |
| Retired & ill health | 1.38 | 0.77, 2.47 | 1.69 | 0.71, 4.02 |
| Retired & involuntary | 0.73 | 0.36, 1.47 | 2.27 ^ŧ^ | 0.90, 5.73 |
| Retired & voluntary | 0.63 | 0.34, 1.17 | 1.07 | 0.41, 2.80 |
| Work & involuntary | 1.05 | 0.56, 2.00 | 0.87 | 0.23, 3.31 |
| Work & voluntary | 0.53* | 0.28, 0.99 | 1.17 | 0.38, 3.60 |
| **Work status & motivation × Time** |  |  |  |  |
| Retired & SPA | Ref |  | Ref |  |
| Retired & ill health | 1.11 ^ŧ^ | 0.99, 1.24 | 0.90 | 0.78, 1.04 |
| Retired & involuntary | 1.09 | 0.96, 1.25 | 0.92 | 0.80, 1.07 |
| Retired & voluntary | 1.10 | 0.98, 1.23 | 0.87 ^ŧ^ | 0.75, 1.02 |
| Work & involuntary | 1.01 | 0.89, 1.15 | 1.14 | 0.93, 1.40 |
| Work & voluntary | 1.02 | 0.90, 1.15 | 0.81* | 0.66, 0.99 |

^ŧ^ p<0.1 *p<0.05 **p<0.01

**Figure S2** Work status beyond SPA and the trajectory of memory for men

**Figure S3** Work status beyond SPA and the trajectory of verbal fluency for women

**Figure S4** Work status beyond SPA and the trajectory of verbal fluency for men

**Figure S5** Work status beyond SPA and the trajectory of depression for women
